# Craniofacial Cephalometric Morphology in Polish Adolescents with Cleft Palate Only

**DOI:** 10.3390/jcm13154507

**Published:** 2024-08-01

**Authors:** Alicja Zawiślak, Barbara Wędrychowska-Szulc, Katarzyna Grocholewicz, Joanna Janiszewska-Olszowska

**Affiliations:** 1 Department of Maxillofacial Orthopaedics and Orthodontics, Institute of Mother and Child, ul. Kasprzaka 17a, 01-211 Warsaw, Poland; 2 Department of Interdisciplinary Dentistry, Pomeranian Medical University, al. Powstańców Wlkp. 72, 70-111 Szczecin, Poland; alicja.zawislak@pum.edu.pl (A.Z.); katarzyna.grocholewicz@pum.edu.pl (K.G.); 3 Private Praxis Vita-Med 2, Ledochowskiego 16/U3A, 71-004 Szczecin, Poland; bwedrychowska@gmail.com

**Keywords:** cephalometric analysis, cleft palate, lateral cephalogram, craniofacial deformity, cephalometry

## Abstract

**Background**: Cephalometric studies indicate that craniofacial morphology in patients with cleft palate only (CPO) differs from other forms of orofacial clefts and healthy patients. Planning orthodontic treatment for patients with different craniofacial deformities requires knowledge on the craniofacial complex. The aim of the present study was to describe the cephalometric craniofacial morphology in adolescents with cleft palate only compared to generally healthy orthodontic patients. **Methods:** The study comprised 100 lateral cephalograms (taken in the years 2003–2020) of Polish patients with cleft palate only aged from 11.1 to 14.2 (mean age 12.43 y) and a matched control group of 100 children without orofacial clefts aged 12–14 (mean age 12.25). All digital images were analyzed in specialized cephalometric software. **Results**: Statistically significantly lower values of both SNA (*p* < 0.001) and ANB (*p* < 0.001) were found in the study group versus the control group. Mandibular line to cranial base angle (ML-NSL) as well as maxillary base to cranial base (NL-NSL) were significantly higher in the CPO group. Both the maxilla and mandible were rotated distally in CPO. Moreover, the intermaxillary vertical angle (ML-NL) was reduced in CPO. Mandibular angle in CPO was significantly higher (*p* = 0.005), reflecting posterior mandibular rotation. **Conclusions**: In adolescents with CPO, maxillary deficiency is found, without a severe sagittal jaw discrepancy, with a slight compensatory lingual inclination of the lower incisors. Mandibular deficiency in CPO is concurrent with posterior rotation and an increased mandibular angle.

## 1. Introduction

Orofacial cleft (OFC) is the second most common birth defect (after phimosis), and it is the most frequent malformation within the craniofacial region. The incidence rate is approximately 1 in 700 live births, although this varies among different ethnic groups. The development of face and palate is a complex process involving the growth and fusion of the primary and secondary palatal shelves. Disruptions in this intricate process can lead to the formation of clefts. Various types of clefts can occur, including cleft lip (CL), cleft palate only (CPO), and cleft lip and palate (CL/P). The severity of the cleft depends on the extent of the structures involved [1,2,3].

The size and location of the cleft significantly impact the function of the orofacial complex, leading to issues with hearing, breathing, speech, and feeding in affected patients. That is why treatment of OFCs is multidisciplinary, requiring the expertise of various specialists, including plastic surgeons, orthodontists, otolaryngologists, speech therapists, audiologists, and pediatricians. This comprehensive approach ensures that all aspects of the condition are addressed, from surgical repair to long-term functional and aesthetic outcomes, as well as support for overall well-being [4].

The etiology of cleft lip and palate is complex, involving both genetic and environmental factors. Genetic predisposition plays a significant role, with environmental influences such as maternal smoking, infectious diseases during pregnancy, poor nutrition, and certain medications acting as modifying factors. Notably, the genetic component of CPO is more substantial than in other types of clefts, highlighting the unique nature of this malformation [2,5,6].

As cleft palate is a phenotypically diverse inborn malformation, its classification is difficult. No uniform classification of clefts has been widely accepted allowing use of universal terminology describing cleft of the palate only. Exploration of the evolution of ideas regarding cleft palate classification includes the schemes described by numerous researchers, including: Fogh-Andersen (1947) (isolated cleft palate), Veau (1931) (cleft of the soft palate, cleft of the soft and hard palate up to the incisive foramen), Womersley and Stone (1987) (cleft palate alone), Kernahan and Stark (1958) (posterior to the incisive foramen), ACPA (Berlin, 1971) (cleft of the hard palate (uranoschisis)), Broadbent (1969) (cleft of the soft palate (staphyloschisis or veloschisis), cleft of the soft palate (uranostaphyloschisis), (cleft of the posterior (secondary) palate), Millard (2017) (cleft palate, cleft palate only), Carroll and Mossey (2012) (HSH (hard, soft) or S (soft)), and Kernahan (1971) (striped Y-diagram) [7,8,9,10,11,12,13,14,15].

The authors of the present study consequently use the term “cleft palate only” (CPO). In various studies, the term “cleft palate” is used for orofacial clefts (Madachi et al., 2017) [16], whereas the term “isolated cleft palate” is both used for CPO and for various non-syndromic clefts [17,18,19,20].

According to previous cephalometric studies, significant differences exist referring to craniofacial morphology in CPO compared to UCLP, BCLP (Diah et al., 2007, Azouz et al., 2020). SNA measurements have revealed notable differences between groups, with the BCLP group exhibiting higher values and the CP group showing lower values in comparison to healthy patients [20,21]. Planning orthodontic treatment and prognosing the eventual need for orthognathic surgery in groups of patients with different craniofacial deformities require knowledge on the characteristics of craniofacial morphology [3].

However, no studies could be found in the literature referring to craniofacial cephalometric morphology in Polish adolescents with CPO.

Adolescence is a time particularly relevant to orthodontic treatment due to unique challenges associated with altered dental arch relationships and the potential for both dentoalveolar compensation in early permanent dentition or preparing for future orthognathic surgery, when orthodontic treatment with fixed appliances ais performed. Thus, identifying specific orthodontic problems is essential for improving proper strategies in this critical period for orthodontic evaluation and intervention. As a working hypothesis, we assumed that craniofacial morphology in CPO differs from that of healthy patients, referring to shorter maxillary length (SNA value), and compensatory incisor protrusion.

The aim of the present study was to compare cephalometric craniofacial morphology (specifically: SNA, SNB, ANB, SNPg, NSBa, GntgoAr, NL-NSL, ML-NSL, ML-NL, H, 1+:1−, 1+:NA, 1−:NB, Nasolabial angle, Pg:NB, Wits, Index) in adolescents with CPO to that of generally healthy young orthodontic patients.

## 2. Materials and Methods

The retrospective study comprised 100 lateral cephalograms (taken in the years 2003–2020) of Polish adolescents with CPO versus a control group (n = 100) recruited from consecutive patients referred for orthodontic treatment. The sample size was verified for SNA angle using an online calculator (select-statistics.co.uk), assuming a 95% confidence level and 80% power to detect a difference of 3 degrees, yielding a sample of 16 participants per group sufficient for comparison to be made.

The inclusion criteria for the study were as follows: patients aged 11–15 years, Polish origin, clear diagnosis of CPO (excluding Pierre-Robin sequence), sufficient quality of the cephalogram to allow identification of selected cephalometric landmarks, and absence of other significant congenital anomalies to exclude the influence of other anomalies on the study results.

The exclusion criteria were as follows: patients younger than 11 years or older than 15 years, as age can impact results due to stage of facial development; patients with other craniofacial disorders; patients who had undergone corrective surgeries in the facial or cranial region, as such surgeries can affect the study results; patients with serious chronic diseases (e.g., metabolic or endocrine disorders), which can influence facial and cranial development; and unclear or incomplete X-ray images, as high-quality images are necessary for accurate measurements.

During the study period, 263 X-ray images were reviewed. However, only 100 of these met the inclusion criteria and were used for the analysis. This ensured that the study data were consistent and allowed for precise comparisons between groups.

All patients from the study group were operated on using a modified Langenbeck method. This is a common surgical technique for cleft palate repair that aims to reestablish proper muscle function by repositioning the palatal muscles. It involves making relaxing incisions and elevating mucoperiosteal flaps to achieve tension-free closure of the cleft, thereby improving velopharyngeal competence and reducing the incidence of oronasal fistulas.

**Figure 1:**
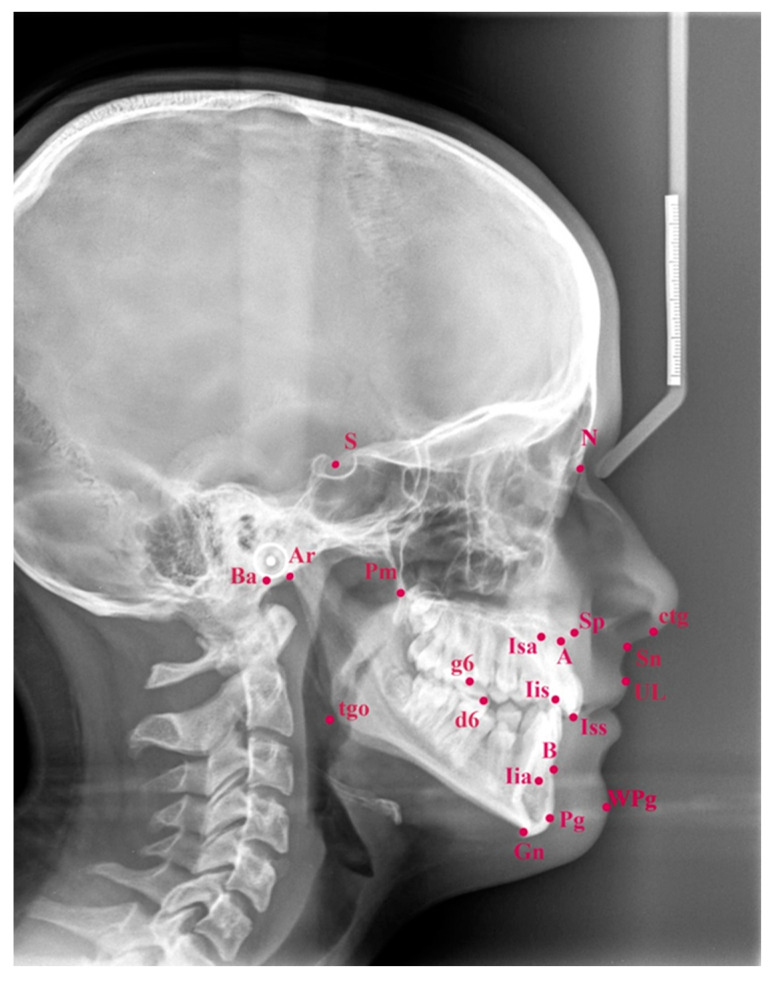
Cephalometric landmarks used for the purpose of this study.

A digital X-ray device (Cranex Tom, Soredex, Tuusula, Finland) was used to perform all cephalograms. The digital X-rays received were analyzed in software designed for cephalometric analysis Ortodoncja 7.0 (Orto-Bajt, Wroclaw, Poland) using the method by Segner and Hasund [22]. Cephalometric landmarks used for the purpose of this study are presented in Figure 1.

Cephalometric variables used and their specific significance in orofacial clefts were presented in a previous study [23]. Table 1 presents reference values used for the analysis.

The alpha (α) level was set at 0.05 as the threshold for statistical significance; the results yielding a *p* < 0.05 were considered statistically significant. The Shapiro–Wilk test was used to verify a normal data distribution. With normal distribution, mean and standard deviation were reported, whereas in other cases non-parametric descriptive statistics were reported, e.g., the median and the first and third quartiles.

Every analysis was performed by two operators and inter-examiner reliability was verified using ICC (Intraclass Correlation Coefficient). A mean value between the two measurements was used for further comparisons and analysis of correlations. Twenty-one randomly selected cephalograms were reanalyzed two weeks later by the same investigators, and ICC (Intraclass Correlation Coefficient) was calculated to assess inter- and intra-examiner reliability. The ICC values were adopted according to Cicchetti et al. [24], as excellent accordance between the measurements with ICC above 0.75, good accordance with ICC between 0.6 and 0.75, fair accordance with ICC: 0.4–0.6, and weak accordance with ICC below 0.4. To evaluate the agreement between two raters, the Intraclass Correlation Coefficient (ICC) was used, utilizing a two-way mixed-effects model.

The comparative analysis of two independent groups was conducted using Welch’s *t*-test for variables with normal distribution, and the Wilcoxon rank-sum test for the other variables. The correlations between numerical variables were quantified using Spearman’s rho. The correlations of the cephalometric variables were visually presented as a correlation matrix.

Analyses were conducted using the R Statistical language (version 4.3.1; R Core Team, 2023) on Windows 10 pro 64-bit (build 19045), using the packages *irr* (version 0.84.1; Gamer M et al., 2019), *rstatix* (version 0.7.2; Kassambara A, 2023), *sjPlot* (version 2.8.15; Lüdecke D, 2023), *report* (version 0.5.7; Makowski D et al., 2023), *ggstatsplot* (version 0.12.1; Patil I, 2021), *gtsummary* (version 1.7.2; Sjoberg D et al., 2021), *MASS* (version 7.3.60; Venables WN, Ripley BD, 2002), *dplyr* (version 1.1.3; Wickham H et al., 2023), and *psych* (version 2.3.9; William Revelle, 2023) [25,26,27,28,29,30,31,32,33].

## 3. Results

During the study period, 263 X-ray images were reviewed. However, only 100 of these met the inclusion criteria and were used for the analysis. The characteristics of the study and control groups referring to age and gender are presented in Table 2.

The evaluation of inter-rater reliability yielded highly reliable results, as evidenced by the ICC values and their respective CI 95%. For all the measurements, excellent inter-examiner reliability was demonstrated according to the method by Cicchetti et al. [25].

The distribution of cephalometric variables in the study and control groups is presented in Table 3.

A statistically significantly lower value of SNA (*p* < 0.001) was found in the CPO group versus the control group. Moreover, ANB was significantly lower (*p* < 0.01) in the study group, reflecting a sagittal maxillary deficiency. Mandibular angle (Gn-tgo-Ar) was significantly higher in the CPO group (*p* = 0.005), indicating a posterior mandibular rotation during growth.

Maxillary inclination to cranial base (NL-NSL) was increased in CPO both referring to the norm and to the control group, reflecting maxillary posterior rotation. Mandibular inclination to cranial base (ML-NSL) was higher in CPO children than in the control group, confirming a tendency to posterior mandibular rotation. Vertical intermaxillary angle (ML-NL) in CPO adolescents was lower than in the control group, indicating a more pronounced rotation of the maxilla compared to that of the mandible.

Nasolabial angle was significantly lower in CPO patients compared to the control group.

No statistically significant difference was found referring to the inclination of upper incisors to NA line, whereas lower incisors in the CPO group were slightly retruded—they had a lower angle of inclination to NB line. Thus, interincisal angle was higher in CPO patients.

WITS appraisal was lower in CPO than in the control group, confirming a sagittal jaw discrepancy. The index of the medium to lower face height did not significantly differ from that in non-cleft controls.

Correlations of cephalometric values with age are presented in Table 4.

Despite a narrow age span within the CPO and control groups, cephalometric variables proved age-dependent.

**Figure 2:**
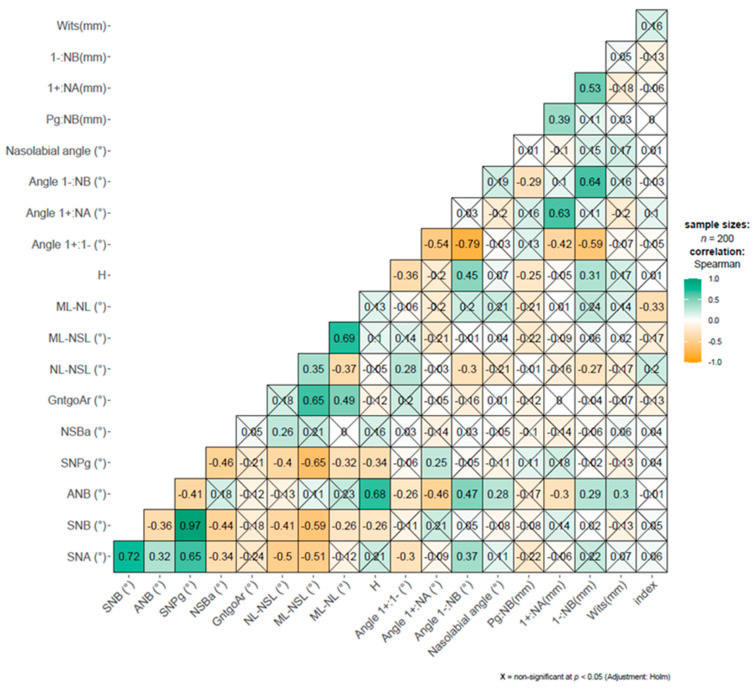
The interrelationship between cephalometric values in the study group.

Correlations between cephalometric values are presented in Figure 2.

The correlations visible reflect the geometric interrelationship between cephalometric values.

## 4. Discussion

This rigorous analysis engaged a study population characterized by CPO counterbalanced by a matched control group free from orofacial clefts. Cephalometric variables including skeletal, dental, and soft tissue parameters can be used to facilitate a recognition of morphological discrepancy and may aid in choosing therapeutic strategies for CPO patients.

The ICC for repeated cephalometric landmark identification on 2D lateral cephalometric radiographs in the literature is 0.8 [34,35]. No studies could be found describing the repeatability of landmark identification in patients with cleft palate. The first and second authors are experienced in the analysis of cephalometric radiographs of patients with clefts; however, values comprising the maxillary base seemed more difficult to identify due to the absence of bony palatal structures. Cephalometric analysis has recently been performed using modern technologies including machine learning and artificial intelligence and promising results were presented referring to accuracy and repeatability of landmark identification [36].

Few studies could be found in the literature describing cephalometric craniofacial morphology of patients with CPO [17,21,37,38,39,40,41,42,43,44]. Moreover, they are difficult to identify in databases of the scientific literature due to diverse use of terminology. A study dealing with cleft infants could be found [45], which does not refer to orthodontic or orthognathic treatment. Studies referring to cephalometric analysis in cleft patients are based on various cephalometric measurements (Table 5).

Due to various cephalometric landmarks and measurements having been used in previous studies, it is difficult to compare the results from different papers. The study groups of the papers found range from 32 to 189 subjects. Mean age in the studies tabularized ranges from 2 months to 21 years. Thus, the population of the present study belongs to the largest and is more homogenous.

Moreover, is difficult to compare the results of the present study to those from other studies, because the conclusions of the previous papers refer to different aspects of cephalometric analysis, including reliability of digital measurements [39], the benefit of 3D cephalometrics [42], comparison of different surgical techniques [40], craniofacial morphology of patients with different clefts [43], and comparison of craniofacial morphology in patients with cleft lip (CL) to those with SCP (submucous cleft palate) or adult patients with different unoperated clefts [21].

Although cephalometric analyses in different studies were made using various landmarks and measurements, most studies use ANB, NSBa, SNB, SNA, and WITS, and those variables could be compared.

The mean ANB angle of e1.39 in the study group is within normal values. Compared to the control group (3.34), it is significantly lower (*p* < 0.001), reflecting a mild sagittal discrepancy resulting from the cleft. On the other side, the mean age of the CPO group is 12.43 years and a possible decrease of ANB with age indicates a potential for worsening sagittal jaw relation but does not prognose a severe discrepancy. This is consistent with results referring to ANB angle in adult CPO patients; the mean value in the CPO group was −1.75 (2.95 in the control group) [23].

Lower SNA values in the CPO group compared to the control group further confirm a maxillary deficiency. A recent study on adult CPO patients [23] reported that SNA was lower in the CPO group, which may be due to the cleft itself and to scarring from palatal surgery [46].

A recent study by Tsuji et al. (2021) [17] describing craniofacial cephalometric morphology in CPO on 5-year-old girls (n = 36) provided findings that are not contradictory to the present study: a bimaxillary retrusion (mean values: SNA = 80.1, SNB = 76, ANB = 4.1) and an increased gonial angle (mean value = 130).

Nasolabial angle (NLA) is significantly lower in CPO, possibly indicating a flattened nasal tip due to a reduced skeletal support. However, no studies analyzing nasal morphology could be found to support this explanation.

The finding of the present study that nasolabial angle was significantly lower in CPO patients compared to the control group may reflect a reduced support of the columella from the retropositioned anterior nasal spine. Moreover, the difference was statistically insignificant in adult CPO patients [23], probably due to thinner soft tissues, additionally reduced by scarring from secondary surgical corrections.

The finding of the present study that lower incisors in the CPO group were retruded compared to the control group reflects dentoalveolar compensation of sagittal intermaxillary discrepancy in CPO. This discrepancy is more pronounced with growth, as in adult CPO patients both the maxillary and mandibular incisors are statistically significantly retruded compared to a control group [23].

It is interesting that ML-NL in the non-cleft group is higher than mean values by Segner and Hasund. Posterior mandibular rotation during growth should be considered a typical feature of craniofacial morphology in mandibular deficiency in CPO, confirming previous findings in adult CPO patients [24]. Since NL-NSL is increased both referring to the norm and to the control group, and the index of the medium to lower anterior face height is normal, this is evidence of decreased posterior maxillary height, similar to adult CPO patients [23].

The present study confirmed correlations of cephalometric values with age reported earlier in the literature referring to both healthy and cleft patients [47].

Craniofacial morphology in CPO depends on age, sex, cleft severity, and surgical procedures [21,40,41,42]. In the study by Parikakis et al. (2018) [40] comparing cephalometric values among two age groups (5-year-old and 10-year-old children) operated on, using the Veau–Wardill–Kilner technique (Nyl én, 1961) and minimal-incision technique (Mendoza et al., 1994), mandibular length was significantly shorter in the minimal-incision group. Referring to SNA angle, in the study by Parikakis et al. (2018) [40], mean SNA angle in four study groups with CPO (depending on cleft severity and surgical technique) ranged from 79.90 to 80.30, which is very similar to the results of the present study (80.10). Timing of hard palatal repair is regarded as an important factor influencing maxillary growth as well [41]. Thus, it is evident that reduced anterior maxillary height is a feature typical for operated patients with CPO. Similarly, in the study by Ye et al. (2015) [41], SNA was significantly lower in an operated cleft lip and palate group (UCLP and CPO) (76.59 ± 4.49) than in an unoperated control group of UCLP and CPO patients (82.07 ± 2.41), as well. However, it has to be noted that the study groups were small (n = 40 each) and non-homogenous. Thus, the SNA values could be lower than in the present study due to inclusion of UCLP patients (characterized by more severe maxillary deficiency).

In the study by Heliövaara et al. (2009) [39] on 7-yearold boys with operated SCP, mean SNA was 79.7 ± 2.8, very similar to the present study. It is noteworthy that the study group was younger than in the present study and was characterized by a low severity of the cleft. In 6-year-old girls with operated CPO, mean SNA was 78.9 ± 3.6 [38], a lower value than in the present study, probably due to the younger age of patients included by Heliövaara et al. (2003) [38].

A possible limitation of the present study refers to manual identifying cephalometric landmarks. An obvious direction for future research is the use of AI tools for further analysis. Future studies should focus on developing more advanced machine learning models, including deep learning techniques, to enhance the accuracy and reliability of cephalometric analysis. Additionally, exploring how AI tools can be implemented into everyday clinical practice to improve efficiency and patient outcomes is essential. Such research could also boost the effectiveness of models across diverse patient groups including severe cleft cases [44,45,46].

Another research area should be the interdisciplinary applications of AI in orthodontics, maxillofacial surgery, and craniofacial studies. Comparative studies are needed to assess the effectiveness of AI technologies compared to traditional-manual methods [44,45,46].

## 5. Conclusions

In adolescents with CPO, maxillary deficiency is present, without evidence of severe sagittal jaw discrepancy, with a slight compensatory lingual inclination of the lower incisors. Mandibular deficiency in CPO is associated with posterior rotation (increased mandibular angle).

## Figures and Tables

**Table 1:** Cephalometric variables used (according to Segner and Hasund, 1998).

Abbreviation	Mean Value	Range	Reference Points or Lines
SNA	82	79–85	Sella-Nasion-A: Sagittal maxillary position referring to cranial base
SNB	80	77–83	Sella-Nasion-B: Sagittal position of the mandibular alveolar part referring to cranial base
ANB	2	0–4	A-Nasion-B: Sagittal relation between the maxilla and mandible
SNPg	82	78–84	A-Nasion-Pg: Sagittal position of the chin referring to cranial base
NL-NSL	8	4–12	Maxillary base-cranial base: Vertical maxillary inclination relative to cranial base
ML-NSL	28	23–33	Mandibular base-cranial base: Vertical mandibular inclination relative to cranial base
ML-NL	20	12–27	Maxillary base-cranial base: Vertical jaw relationship
NS-Ba	132	128–136	Inclination of the clivus to cranial base
Gn-tgo-Ar	122	115–129	Gonial angle
H	9	6–12	Upper lip–soft-tissue chin–NB line: Angle between the line of the upper lip and soft-tissue chin relative to line NB—inclination of the soft tissue profile
1+:1−	133	125–141	Angle between the long axes of upper and lower central incisors
1+:NA	21	18–25	Upper incisor inclination to NA line
1+:NB	24	20–28	Lower incisor inclination to NB line
Nasolabial angle	110	103–117	Nasal base–upper lip: Angle between nasal base and upper lip
Index	80	73–87	Proportion between the upper and lower face height (in percentage)
Pg:NB (mm)	2.3	0.3–4.3	Distance Pg–NB line: Describes chin prominence
1+:NA (mm)	3.7	1.7–5.7	Distance: Incisal edge of the upper central incisor–NA line
1−:NB (mm)	3.8	−1.2–8.8	Distance: Incisal edge of the lower central incisor–NB line
Wits (mm)	0	−2–2	Distance: Perpendicular projections of points A and B on the occlusal plane

**Table 2:** Characteristics of the study sample.

Characteristic	Total Cohort	Group		*p* Wilcoxon Rank-Sum Test
	Mean (Q1, Q3)	CPO, n = 100	Control, n = 100	
Age, years	12.42 (11.61, 14.10)	12.43 (11.11, 14.24)	12.25 (12.00, 14.00)	0.505
Gender				
girls	100.00 (50.00%)	50.00 (50.00%)	50.00 (50.00%)	
boys	100.00 (50.00%)	50.00 (50.00%)	50.00 (50.00%)	

**Table 3:** Cephalometric variables of the study and control groups.

Cephalometric Variable (Unit)	Total Sample n = 200	Group		*p* (Welch’s *t*-Test) ^1^ *p* (Wilcoxon Rank-Sum Test) ^2^
		CPO, n = 100	Control, n = 100	
	Mean (SD) ^a^ Median (Q1, Q3) ^b^	Mean (SD) ^a^ Median (Q1, Q3) ^b^	Mean (SD) ^a^ Median (Q1, Q3) ^b^	
SNA (°)	79.07 (4.42) ^a^	77.70 (4.93) ^a^	80.44 (3.34) ^a^	**<0.001** ^1^
SNB (°)	76.71 (4.53) ^a^	76.31 (5.07) ^a^	77.11 (3.90) ^a^	0.217 ^1^
ANB (°)	2.37 (3.33) ^a^	1.39 (3.42) ^a^	3.34 (2.95) ^a^	**<0.001** ^1^
SNPg (°)	77.47 (4.57) ^a^	77.19 (5.10) ^a^	77.75 (3.99) ^a^	0.395 ^1^
NSBa (°)	130.69 (6.00) ^a^	130.71 (6.60) ^a^	130.68 (5.36) ^a^	0.964 ^1^
GntgoAr (°)	128.90 (7.43) ^a^	130.38(5.36) ^a^	127.42 (7.30) ^a^	**0.005** ^1^
NL-NSL (°)	11.96 (5.30) ^a^	15.40 (4.78) ^a^	8.52 (3.12) ^a^	**<0.001** ^1^
ML-NSL (°)	36.19 (6.90) ^a^	37.53 (7.52) ^a^	34.85 (5.95) ^a^	**0.006** ^1^
ML-NL (°)	24.24 (7.05) ^a^	22.14 (7.34) ^a^	26.34 (6.10) ^a^	**<0.001** ^1^
H	11.27 (5.89) ^a^	10.52 (5.74) ^a^	12.01 (5.98) ^a^	0.074 ^1^
1+:1 (°)	131.00 (11.67) ^a^	134.91 (11.64) ^a^	127.09 (10.37) ^a^	**<0.001** ^1^
1+:NA (°)	23.15 (8.22) ^a^	23.29 (8.97) ^a^	23.01 (7.44) ^a^	0.810 ^1^
1−:NB (°)	24.68 (18.78, 28.29) ^b^	21.50 ^b^ (16.01, 25.11)	26.75 ^b^ (23.38, 30.10)	**<0.001** ^2^
Pg:NB (mm)	1.05 (0.20, 2.56) ^b^	1.10 (0.39, 1.96) ^b^	0.95 (0.00, 6.45) ^b^	0.587 ^2^
1+:NA (mm)	2.45 (1.20, 5.41) ^b^	2.55 (1.11, 3.80) ^b^	2.40 (1.28, 19.23) ^b^	**0.006** ^2^
1−:NB (mm)	2.40 (1.30, 5.43) ^b^	1.80 (0.83, 3.43) ^b^	3.00 (1.70, 20.18) ^b^	**<0.001** ^2^
Wits (mm)	0.80 (−3.29, 0.96) ^b^	−1.65 (−3.29, 0.20) ^b^	−0.05 (−3.10, 2.10) ^b^	**0.010** ^2^
Nasolabial angle (°)	111.90 (103.66, 119.33) ^b^	106.85 (98.81, 116.48) ^b^	114.90 (108.28, 122.08) ^b^	**<0.001** ^2^
Index	82.18 (75.88, 86.18) ^b^	81.65 (74.50, 86.24) ^b^	82.30 (78.30, 86.10) ^b^	0.368 ^2^

**Table 4:** Correlations between cephalometric variables and age in the study (CPO) and control groups.

Variable	Correlations between Cephalometric Variables and Age	
	Study Group	Control Group
	Spearman’s Correlation Coefficient	
SNA [°]	r = −0.018, *p* = 0.776	r = 0.062, *p* = 0.317
SNB [°]	r = 0.179, *p* = 0.004 *	r = 0.172, *p* = 0.005 *
ANB [°]	r = −0.251, *p* < 0.001 *	r = −0.165, *p* = 0.007 *
SNPg [°]	r = 0.246, *p* < 0.001 *	r = 0.232, *p* < 0.001 *
NSBa [°]	r = −0.013, *p* = 0.838	r = −0.049, *p* = 0.428
GntgoAr [°]	r = −0.2, *p* = 0.001 *	r = −0.125, *p* = 0.043 *
NL-NSL [°]	r = −0.207, *p* = 0.001 *	r = −0.015, *p* = 0.805
ML-NSL [°]	r = −0.132, *p* = 0.032 *	r = −0.148, *p* = 0.016 *
ML-NL [°]	r = 0.044, *p* = 0.481	r = −0.132, *p* = 0.033 *
H	r = −0.113, *p* = 0.068	r = −0.242, *p* < 0.001 *
1+:1− angle [°]	r = −0.245, *p* < 0.001 *	r = 0.084, *p* = 0.174
1+:NA angle [°]	r = 0.287, *p* < 0.001 *	r = 0.004, *p* = 0.95
1−:NB angle [°]	r = 0.155, *p* = 0.012 *	r = −0.019, *p* = 0.764
Nasolabial angle [°]	r = −0.204, *p* = 0.001 *	r = −0.144, *p* = 0.02 *
Pg:NB [mm]	r = 0.374, *p* < 0.001 *	r = 0.086, *p* = 0.166
1+:NA [mm]	r = 0.474, *p* < 0.001 *	r = −0.028, *p* = 0.654
1−:NB [mm]	r = 0.22, *p* < 0.001 *	r = −0.072, *p* = 0.246
Wits [mm]	r = −0.097, *p* = 0.117	r = −0.057, *p* = 0.357
Index	r = 0.026, *p* = 0.675	r = −0.186, *p* = 0.002 *

* Statistically significant (*p* < 0.05).

**Table 5:** Studies on cephalometric analysis in children with CPO (from most recent to oldest).

Author	Year	Characteristics of Subjects					Cephalometric Measurements
		Number of Subjects	Origin	Gender	Mean Age (Age Range)	Characteristics of Malformation	
Tsuji K et al. [17]	2021	77	Japanese	F	5.36 (±0.36)	CPO	Facial angle, AB plane, Y-axis, FH to SN, SNA, SNB, ANB, NPog to SN, nasal floor to SN, nasal floor to FH, mandibular plane to SN, mandibular plane to FH, ramus plane to SN, ramus plane to FH, gonial angle, U1 to SN, U1 to FH, L1 to mandibular plane, interincisal angle, occlusal plane to SN, occlusal plane to FH, N-S, N-ANS, ANS-Me, N-ME, S’-Ptm’, A’-Ptm’, Ptm’-Ms, A’-Ms, Is-Is’, Mo-Ms, Is-Mo, Gn-Cd, Pog’-Go, Cd-Go, Ii-Ii’, Mo-Mi, Ii-Mo
Da Silva F. et al. [37]	1993	61	Latin American	F, M	11 months (M) 2 months (F)	CPO	SN, Go-Gn, Co-Go, Co-Gn, P-NB, CoGoMe, S-Go, N-Me, ANS-Me, SNB, SNGoGn, SNGn
Heliövaara A. et al. [38]	2003	93	Caucasian	F	6.2 (range 5.5–7.5)	CPO	NSBa, NS, SBa, NBa, ANA, SNB, ANB, SNPog, ANS-Me, N-ANS, S-Go, GN-CD, Me-GO, Ar-Go, NSL-ML, NL-NSL, Sna, Snb, anb, Snpog, n-sn-pog, n-prn-pog, cm-sn-ls
Diah E et al. [21]	2007	92	Indian	F, M	21.6 (range 16–47 years)	UCL, UCLP, BCLP, CPO	SNA
Lu. D.W. et al. [43]	2007	107	Chinese	F, M	7.5 years 7.8 years 7.2 years 7.3 years	TCS PRS CLP CPO	SNA, SNB, ANB, MP-SN, OP-SN, Y-axis, PP-FH, PP-MP, BaNPtGn, ArGoMe, AGNH-Go-Me, NMe, N-ANS, ANS-Me, ANS-Pt, S-Pt, Cd-Go, Pog-Go, AGNH-AGNH’, S-Go, S-Go/N-Me, ANS-Me/N-Me
Heliövaara A. et al. [39]	2008	32	Caucasian	M	6.8 (range 5.5 to 8.6 years)	SCP	NSBa, SNA, SNB, ANB, SNPg, ANS-PM, N-ANS, ANS-ME, S-GO, NSL-ML, GN-CD, ME-GO, Ar-GO, Sna, Snb, anb, BA-PM, ad1-PM, ad1-BA, ad2-PM, ad2-so, pas, PM-u, HY-HY’
Antonarakis G.S. et al. [42]	2015	189	Caucasian, Asian, African	F, M	Minimum age of 15 years	PRS excluded	Maxillary length, maxillary protrusion, maxillary height, maxillary inclination
Ye B. et al. [41]	2015	80	Chinese	F, M	19.3 (range 16 to 33 years) 19.7 (range 17 to 31 years)	UCLP without palate repair UCLP with lip and palate repair	SNA, NA-FH, facial angle, SNB, AntCranBase, GoMe/SN, MP-FH, MP-SN, ANB, Y-axis, %Nose, NAAGn, Pog-NB
Parikakis K. et al. [40]	2018	170	Caucasian	F, M	5 (mean age ± 0.6 years) and 10 (mean age 10.3 ± 0.6 years)	PRS excluded	NSBa, SNA, ANB, NSL-NL, NSL-ML, ML-RL, NApg, palatal plane length, mandibular length, n-sp’/n-gn (%), posterior upper FH, posterior FH, facial convexity (G-Sn-Pg’)

CPO—cleft palate only, TCS—Treacher–Collins syndrome, PRS—Pierre–Robin sequence, CLP—cleft lip and palate, UCLP—unilateral cleft lip and palate, SCP—submucous cleft palate.

## Data Availability

All data are available from corresponding author on reasonable request.
